# Supplementary material for: The Systems Biology Research Tool: evolvable open-source software
Source: BMC Syst Biol. 2008 Jun 29;2:55. doi: 10.1186/1752-0509-2-55 (PMC2446383; doi:10.1186/1752-0509-2-55)
Supplement: Additional file 1 — SBRT Archive. An archive of the current version of the Systems Biology Research Tool. [file 1752-0509-2-55-S1.zip › sbrt-1.4.0/doc/developers_guide/api/sbrt/shell/io/FileLineIterator.html]

FileLineIterator


|  |  |  |
| --- | --- | --- |
| **Overview** \| **Package** \| **Class** \| **Use** \| **Tree** \| **Deprecated** \| **Index** \| **Help** | |  |
| **PREV CLASS**   **NEXT CLASS** | **FRAMES**    **NO FRAMES**     **All Classes** |  |
| SUMMARY: NESTED \| FIELD \| CONSTR \| METHOD | DETAIL: FIELD \| CONSTR \| METHOD |  |


---


## sbrt.shell.io Class FileLineIterator<E>

```
java.lang.Object
  sbrt.shell.io.FileLineIterator<E>
```

**Type Parameters:**: `E` - the returned type.

**All Implemented Interfaces:**: java.util.Iterator<E>

**Direct Known Subclasses:**: SerializedFileIterator, TextFileLineIterator

---

``` public abstract class FileLineIterator<E> extends java.lang.Object implements java.util.Iterator<E> ```

This class is used to iterate through the lines of a file.

**Author:**
:   This class was written and documented by
    Jeremiah Wright while in the Wagner lab.

---

**Constructor Summary**

`FileLineIterator()`


| **Method Summary** | |
| --- | --- |
| `protected static void` | `checkFile(java.lang.String fileName)`             Ensures the provided string is the name of a valid file. |

**Methods inherited from class java.lang.Object**

`clone, equals, finalize, getClass, hashCode, notify, notifyAll, toString, wait, wait, wait`

**Methods inherited from interface java.util.Iterator**

`hasNext, next, remove`

**Constructor Detail**

### FileLineIterator

```
public FileLineIterator()
```


**Method Detail**

### checkFile

```
protected static void checkFile(java.lang.String fileName)
```

:   Ensures the provided string is the name of a valid
    file.

    :   **Parameters:**: `fileName` - the name of the file to check for validity.


---


|  |  |  |
| --- | --- | --- |
| **Overview** \| **Package** \| **Class** \| **Use** \| **Tree** \| **Deprecated** \| **Index** \| **Help** | |  |
| **PREV CLASS**   **NEXT CLASS** | **FRAMES**    **NO FRAMES**     **All Classes** |  |
| SUMMARY: NESTED \| FIELD \| CONSTR \| METHOD | DETAIL: FIELD \| CONSTR \| METHOD |  |


---
